# First person – Vincenzo Torraca

**DOI:** 10.1242/dmm.050683

**Published:** 2024-01-26

## Abstract

First Person is a series of interviews with the first authors of a selection of papers published in Disease Models & Mechanisms, helping researchers promote themselves alongside their papers. Vincenzo Torraca is first author on ‘ Transcriptional profiling of zebrafish identifies host factors controlling susceptibility to *Shigella flexneri*’, published in DMM. Vincenzo conducted the research described in this article while he was a Marie Skłodowska-Curie Postdoctoral Fellow at Imperial College London, UK and an ISSF (Institutional Strategic Support Fund)-Wellcome Fellow at the London School of Hygiene and Tropical Medicine, UK, where most of the work was carried out in Prof. Serge Mostowy's lab. He has just started his own group at King's College London, investigating host-pathogen interactions and antimicrobial resistance for globally relevant bacterial pathogens, such as *Shigella* and *E. coli*, using zebrafish as an *in vivo* model.


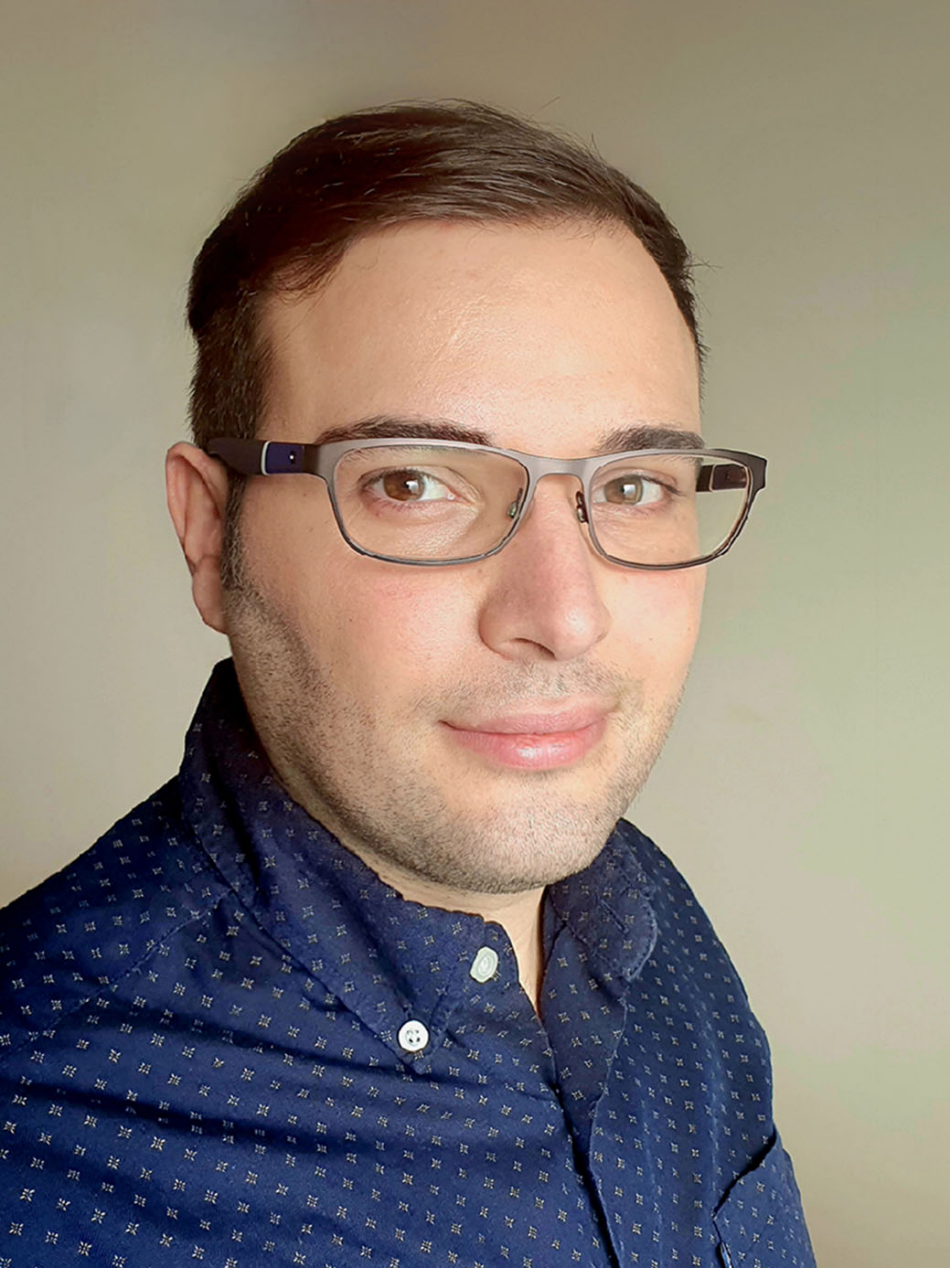


**Vincenzo Torraca**

**How would you explain the main findings of your paper to non-scientific family and friends?**

*Shigella* is an important human pathogen, closely related to *E. coli*. It usually spreads through contaminated water and food, although sexual transmission is also becoming frequent. The immune response against *Shigella* is not fully understood and a clear picture of the global host response against *Shigella* could help identify new therapeutic options. In our article, we determined which host genes are induced after *Shigella* infection. We found that many innate immune processes are activated during the early (i.e. acute) infection phase; however only a few of these genes remain consistently ‘turned on’ during the late (i.e. clearing) infection phase. This allowed us to identify previously unreported factors involved in *Shigella* infection control, such as the receptor Gpr84 and the enzyme Acod1. Zebrafish larvae without a functional *gpr84* or *acod1* gene became more susceptible to *Shigella* infection, suggesting that these factors could represent new therapeutic targets against *Shigella.*

[…] multidrug-resistant *Shigella* is on the rise, and it is becoming increasingly more difficult to find a therapeutic solution for patients.

**What are the potential implications of these results for your field of research?**

We lack a licensed vaccine against *Shigella*, so we rely on antibiotics to treat severe cases of *Shigella* infection. Unfortunately, multidrug-resistant *Shigella* is on the rise, and it is becoming increasingly more difficult to find a therapeutic solution for patients. Our results have identified new genes and pathways controlling the host susceptibility to *Shigella* infection. This may help to guide the development of new therapeutic approaches to control *Shigella* infections.

Our work also showcases the great potential of combining transcriptional profiling and gene editing techniques in the tractable zebrafish model, setting a precedent for similar studies with other important human pathogens. This could be especially valuable for the rapid characterisation of newly emerging pathogens in the future.

**What are the main advantages and drawbacks of the experimental system you have used as it relates to the disease you are investigating?**

The zebrafish larval model is ideal for studying the interaction of pathogens with innate immune cells (i.e. macrophages and neutrophils). Zebrafish are also highly fecund and amenable to genetic manipulations. Combined, these characteristics allowed us to perform gene expression analysis upon *Shigella* infection at a whole-organism level and pursue in depth *Shigella*-inducible candidates to validate their role in controlling susceptibility to infection.

The main drawback of the zebrafish model to study *Shigella* infection is that this system does not allow a complete recapitulation of the human disease. For example, bacteria are not delivered orally and do not transit through the gastrointestinal tract. The initial interactions of *Shigella* with intestinal epithelial cells are ‘bypassed’ by direct delivery of the bacteria to the bloodstream or body cavities, such as the hindbrain ventricle.

**What has surprised you the most while conducting your research?**

The most surprising finding in this research was that the host transcriptional response to *Shigella* changes very rapidly during the course of infection. When comparing the acute and the clearing phases of infection, we found many immune-related genes that were significantly induced exclusively during one or the other phase. Ultimately, comparing these two distinct stages of infection proved valuable, as it allowed us to restrict our investigations to a small pool of genes that remained consistently induced, irrespective of the phase of infection.

**Figure:**
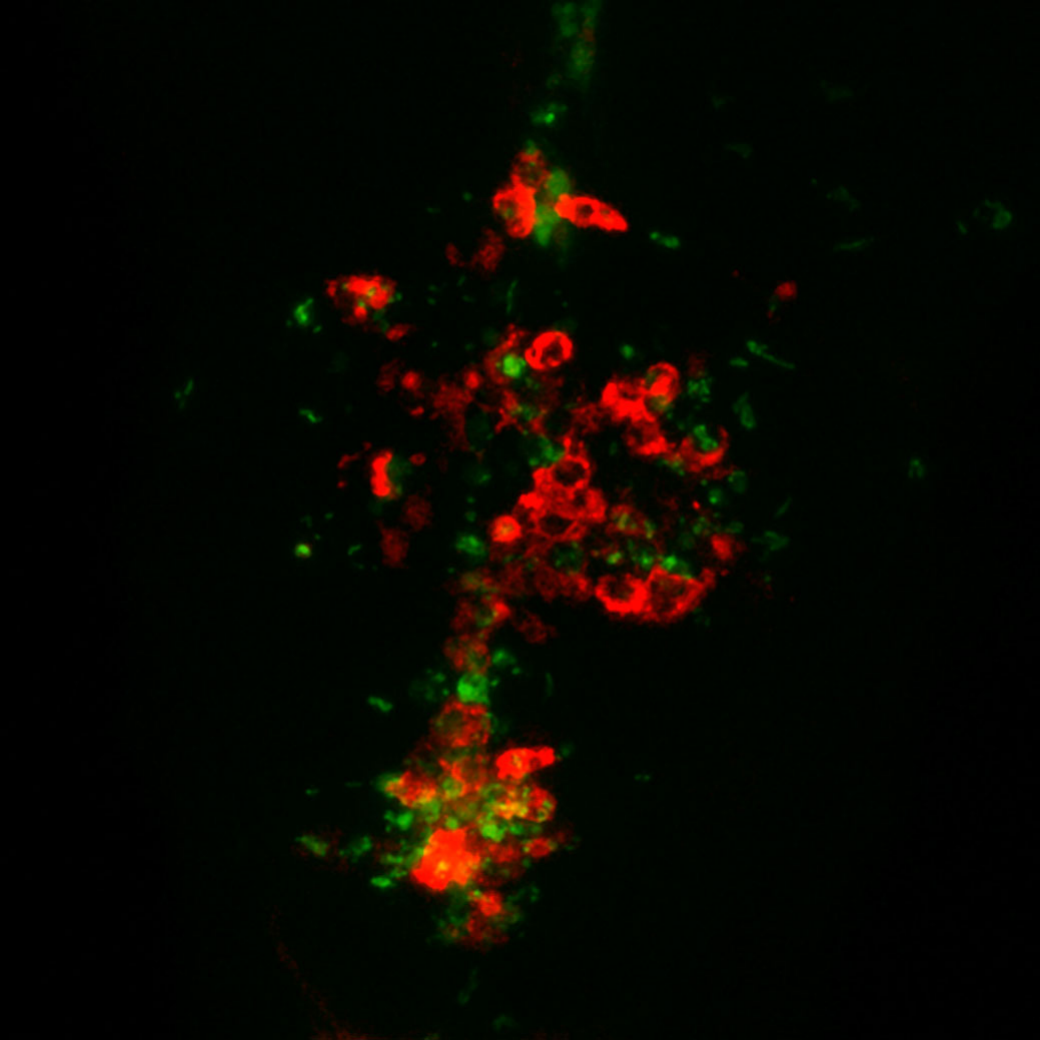
mCherry-labelled macrophages interacting with GFP-labelled *Shigella flexneri* in the hindbrain ventricle of zebrafish larvae.

**Describe what you think is the most significant challenge impacting your research at this time and how this will be addressed over the next 10 years**

There are four serogroups of *Shigella* (referred to as *S. flexneri*, *S. sonnei*, *S. dysenteriae* and *S. boydii*), encompassing numerous serotypes and lineages. There have been recent shifts in the prevalence of different *Shigella* lineages on a global scale and we are identifying remarkable differences in the host response and disease manifestations among different *Shigellae* that could help explain these epidemiological changes. For example, we recently discovered that *S. sonnei* and *S. flexneri* serotypes 2a and 3a (which are highly prevalent worldwide) are also capable of establishing persistent infections *in vivo*, while *S. flexneri* serotype 5a (which has become very rare) is unable to persist. In the future, I plan to exploit the zebrafish model further to comparatively study clinical isolates of different *Shigella* lineages circulating today and identify the factors involved in their success as a pathogen. I envisage that studying the diversity ‘within the same pathogen’ and how this affects disease outcomes will be a key objective of investigations in the next 10 years.

There are insufficient opportunities for scientists' continuous professional development, and more should be created at the institutional, national and international levels.

**What changes do you think could improve the professional lives of scientists?**

It is difficult for early career scientists to stay current and keep up with the latest technological advancements and emerging techniques. Something that is really missing is an acknowledgement that scientists require sufficient dedicated time and resources to develop new skills and introduce new technologies and techniques in their research as they become available. There are insufficient opportunities for scientists' continuous professional development, and more should be created at the institutional, national and international levels.

**What's next for you?**

I recently started my lab at King's College London (KCL), within the Department of Infectious Diseases. At KCL, I will continue to work on enterobacterial pathogens (*Shigella* and *E. coli*) in the zebrafish model, especially focusing on how these pathogens establish persistent and antibiotic-tolerant infections. Persistent infections are not fully cleared and represent an important public health issue because they are difficult to diagnose and treat. They can become asymptomatic and carriers can facilitate dissemination from person to person. Persistent infections are also difficult to eradicate with antibiotics, even when the pathogen causing the infection is not inherently antibiotic resistant. How persistent infections are established and how they become tolerant to antibiotics is not fully understood, and dissecting these processes may help us develop new infection control strategies.

**Is there anything else you would like to add?**

Publishing this article in DMM holds a special significance for me. In 2024, I celebrate 10 years since my first publication as a first author, and that article was also published in DMM (Torraca et al., 2014). Back then, that publication marked my first contribution to research as a novice PhD student. This new publication in DMM marks another key moment in my career, as I take my first steps as a young group leader.
